# Perspectives on utilization of community based health information systems in Western Kenya

**DOI:** 10.11604/pamj.2017.27.180.6419

**Published:** 2017-07-06

**Authors:** Otieno Careena Flora, Kaseje Margaret, Kaseje Dan

**Affiliations:** 1 Tropical Institute of Community Health and Development, Great Lakes University of Kisumu, Kenya

**Keywords:** Community based information systems, dialogue, decision making

## Abstract

**Introduction:**

Health information systems (HIS) are considered fundamental for the efficient delivery of high quality health care. However, a large number of legal and practical constraints influence the design and introduction of such systems. The inability to quantify and analyse situations with credible data and to use data in planning and managing service delivery plagues Africa. Establishing effective information systems and using this data for planning efficient health service delivery is essential to district health systems' performance improvement. Community Health Units in Kenya are central points for community data collection, analysis, dissemination and use. In Kenya, data tend to be collected for reporting purposes and not for decision-making at the point of collection. This paper describes the perspectives of local users on information use in various socio-economic contexts in Kenya.

**Methods:**

Information for this study was gathered through semi-structured interviews. The interviewees were purposefully selected from various community health units and public health facilities in the study area. The data were organized and analysed manually, grouping them into themes and categories.

**Results:**

Information needs of the community included service utilization and health status information. Dialogue was the main way of information utilization in the community. However, health systems and personal challenges impeded proper collection and use of information.

**Conclusion:**

The challenges experienced in health information utilization may be overcome by linkages and coordination between the community and the health facilities. The personal challenges can be remedied using a motivational package that includes training of the Community Health Workers.

## Introduction

Health Information Systems (HIS) are considered fundamental to the efficient delivery of high quality health care. However, a large number of legal and practical constraints influence the design and introduction of such systems [1]. A major issue facing Africa is inability to quantify and analyze the situation it faces with credible data and to use the information in planning and managing service delivery [2, 3]. Establishing good information systems is essential to District Health Systems (DHS) performance improvement in sub-Saharan Africa. The DHS persists in poor performance in spite of decades of efforts to improve it. The hypothesis is that “poor performance is caused by inability to implement health systems improvement policies and strategies as a result of deteriorating socio-economic situations, made worse by inadequate information systems for evidence-based management of the health system” [4]. In terms of data use for local planning and management, countries have not adequately supported health workers who are responsible for collecting data. Some observers [5] speculate that facilitating greater local use of data could improve the overall quality of data, as those collecting the data should be more motivated. Using a simple health care information system that is managed from the lowest level of the health system, for health sector reform and health system management helps to make data more user friendly for local use [6]. Information from Demographic Surveillance Sites (DSS) fed into the district health system for planning and resource allocation based on burden of disease is effective in improving the performance of the service system as well as health outcomes [7].

Most ministries of health across Africa operate some form of Health Management Information System (HMIS) as their primary instrument for generating health system statistics, often representing the majority of national expenditure on health data [8]. Even though the HMIS is the most suitable way of monitoring a wide range of health indicators and in view of the considerable amount of resources invested in their design and implementation, the use of this data to generate statistics for use by decision makers is still extremely limited [9]. The endemic under-use of hugely expensive HMIS data represents an unacceptable inefficiency in already resource-constrained health systems and can be attributed largely to the perceived unreliability of these data, due primarily to poor data coverage [10]. Kenya continues to face health threats characterised by high incidences of infectious diseases such as malaria, high levels of infant mortality (73/1000) and maternal mortality (488/100,000), low levels of life expectancy and deteriorating healthcare facilities [11]. The potential for effective information use has not been fully harnessed systematically to bring about improvements in the health care service delivery. Some known and assumed barriers include: lack of physical access, lack of awareness of what is available; lack of relevance of available information (i.e. not meeting peoples' needs in terms of scope, style or format); lack of time and incentives to access information; and lack of interpretation skills. These problems have also been attributed to “information systems and services, which are not understood, unmanaged and under resourced” [12]. The overall aim of the study was to find out the perspectives on community based information needs and use among health workers and stakeholders in western Kenya. The specific objectives were to: characterize the information needs and information seeking behaviour of the health professionals; assess the knowledge, attitude and practice of health workers on information use; determine the potential challenges and prospects of utilizing health information.

## Methods

This was a qualitative study carried out in 2012.

**Data collection**: The material for this study was gathered through key informant interview guides. The interviewees were purposefully selected from various public health facilities in western Kenya, namely, four facilities in Butere and two in Nyalenda. In total, six health facility in-charges, including three Community Health Extension Workers (CHEWs), thirty three Community Health Workers (CHWs) and eight Community Health Committee (CHC) members were interviewed. The interviews were recorded using a tape recorder. They were later double transcribed by two independent transcribers to ensure the meaning was maintained.

**Data analysis**: A thematic analysis was done to identify the common recurring issues and the main themes were identified that summarised all the views that were col¬lected. This involved an analysis of each question, noting key remarks, concepts or categories, cross-referenced to interview occurrences (interviewee number, interview question and field notes). Cross-case coding of each question in the interview schedule meant that all the data in each question and from each interview was covered exhaustively. A coding scheme was used to describe the perspectives of the health workers and stakeholders. A narrative analysis was also carried out to examine done to look within each case, so that the “story” of an individual's perspective was not lost. Content analyses was applied. The coding frame was prepared a priori, where the categories were established prior to the analysis based upon the objectives of the study. The categories were then agreed on by the principle investigator and one of the researchers and the coding was then applied to the data. Revisions were made as necessary and the categories were tightened up to the point that maximized mutual exclusivity and exhaustiveness [13] and then grouped into emerging themes. There was no restriction concerning the number of codes assigned to a segment of text. The codes were collected into themes which had emerged from the interviews and these themes constituted the different sections in the results.

## Results

**Perceptions of health facility in-charges on need for health information**: The needs for information in the communities were for dialogue and surveillance in order to improve health outcomes and increase service utilization. The main actors in the community who needed information were CHWs, CHEWs and health facility staff. The facility in-charges in both sites (Butere, Nyalenda) agreed that CHWs needed data for dialogue in order to help in the improvement of health outcomes such as antenatal care and immunization (Table 1). However, the sites had different views on when the information was needed and for what purposes. In the Nyalenda facilities, no Community Based Health Information System (CBHIS) existed that was linked to the health facility system. One of the facilities in this site relied on existing surveillance forms from the public health office. The facility only forwarded the information from the community and did not use it locally for planning as illustrated by a quote from a staff member: “The community based tools were introduced just the other day, maybe it (CBHIS) will bring some changes because it has not picked up, it has not picked up at all”. The health facility in-charge in the second facility in the same site voiced similar sentiments: “There has not been much communication and report between the community and the health unit. I think it is because it is still new, so the information system has not been that straight forward the way it is supposed to be. We are still trying to make it streamlined so that it can reach the community”. All respondents mentioned increased uptake of health services as arising from use of information and some changes in health outcomes. Respondents mentioned that these changes were mainly due to increased uptake of available services in the health facilities, improvement in infant mortality and environmental health.

**Table 1:** Perceptions of health facility in-charges on need for health information

	Butere (n=4)	Nyalenda (n=2)
Who needs the information	Health facility management committees	Community Health Workers
	Health facility in-charge	
	Community Health Workers	
When is the information needed	For dialogue during health days	For monitoring disease outbreaks in the community (surveillance reports)
	For defaulter tracing of immunization and tuberculosis	
	For planning on health status improvement	
	For identifying weak areas in service delivery	
For what purpose	Improvement of Antenatal Care (ANC) coverage	Improvement of immunization, malaria outbreaks, latrine coverage
Outcome of information use	Improvement of immunization coverage, ANC attendance	Linkage between facility and community

*Information sources*: all categories of respondents cited the ministry of health as a major source of health information, be it the health facility-based data in the form of facility registers or data at the community level. Other notable sources of data for community dialogue the Community Based Health Information System (CBHIS) which included the CHW log-book, the household register and the assistant chief's register. The data collected was mainly used for dialogue.

*Dialogue topics*: the information needs of the community were mainly on service utilization and health status of the community. Respondents mentioned dialogue as the main way they used information where dialogue led to planning and action in the community most of the issues discussed during dialogue did not originate from the CBHIS, implying that other sources of information were also used for discussions. Some stakeholders disagreed with decisions made during dialogue sessions making it difficult to reach consensus. Topics discussed during the last dialogue session were on general health issues and did not originate from the CBHIS. All categories of respondents mentioned HIV and AIDS as topics mentioned in most dialogue sessions.

*Use of Information*: data was viewed as important for improving the health status of communities and for influencing knowledge and attitudes of mothers on health practices, e.g on sanitation. Community dialogue presented opportunities for using data to inform the discussions and resolutions. All respondents were aware of the appropriate time to use information which is immediately after collection and to analyse the information for use in dialogue with communities, for planning and for implementing health interventions.

However the CHWs who should have been in the frontline of data utilization admitted to not using the information as frequently as expected, with most reporting that they used information more than a month after data collection. At the time of the survey, the community health committees on average had used data in the last one month. Specifically, depending on the topic or health issue being addressed, they had used information between one week and one month after collecting the data. In contrast, the CHEWs and CHWs had used information after a month or more after collection. All the categories of respondents agreed that CHWs should be the ones to use the collected health information in community dialogue, contrary to the CHWs themselves who thought that any trained health officer at a facility level should authorise and also use the information. All categories thought that the ministry of health officers were the ones to decide on when to use data.

However, the CHEWs included the Community Health Committee and the CHWs as decision-makers on when to use data (Table 2 ). The CHWs themselves did not think they can also make decisions on information use. This lack of clarification on decisions on information utilization signifies a disconnect which might be explained by the fact that the CHWs thought that the health officers in facilities or the ministry of health were the decision makers on when to use information. The availability of information and awareness among respondents of how, when and for what purposes information should be used to conduct dialogue in the community implies that the health workers and stakeholders interviewed know and are aware of their responsibility in data utilization (Table 3).

**Table 2:** Knowledge on use of information for dialogue to facilitate continuous improvement of health service delivery (data sources, dialogue)

	Community Health Committee (member)(n=8)	Community Health Workers(n=33)	Community Health Extension Workers (n=3)
Current sources of data for dialogue & decision making	Church	Ministry of Health (Community Based Health Information System)	Household register, log book, chalk board
	Facility (HMIS/CBHIS)	Kisumu Urban Apostolate Program , non-governmental organisation	
Appropriate sources of data for dialogue	Government health facility	From Ministry of Health collected by Community Health Workers.	Household register
	Church health facility	Assistant chief’s registers	Log book
		Health facility registers.	Chalkboard.
Who conducts dialogue?	Community Health Workers	Health officer	Community Health Workers
	Nurse (church health facility – for home based care)	Community Health Workers	Community Health Extension Workers
		Trained health actors	Community Health Committee
			Health facility staff
Who should conduct dialogue	CHWs	Health officer	CHEW
		CHWS	Community Health Committee
		Nurses,	
		Public health officers	
Methods for dialogue	Discussion (brings togetherness in the community)	Teaching and discussions (effective since everyone participates)	Discussions at health facilities and with communities
	Question and answer	Home visits (reaches everyone)	
		Discussions among Support Groups	
Topics discussed during dialogue	HIV/AIDS & related diseases (used information more than 1 month ago)	Cholera (used information more than 1 month ago)	Reproductive Health, Family Planning, Nutrition, Water and Sanitation(used information more than a month ago)
	General health issues (used information within 1 week)	Environmental safety. (used information more than 1 month ago)	
	Support for HIV patients (used information within 1 week)	HIV/AIDS (used information from 1week to 1 month ago)	
	Immunization of 12 to 23 month-old babies.	Health issues (used information more than 1 month ago)	
		Maternal and child health (used information more than 1 month ago)	
		Breastfeeding, hygiene, nutrition (used information more than 1 month ago)	

**Table 3:** Knowledge on use of information for dialogue to facilitate continuous improvement of health service delivery (decision making, data use)

	Community Health Committee (member)(n=8)	Community Health Workers(n=33)	Community Health Extension Workers (n=3)
Decision-maker on when to use information	Nurse	Health Officer (Ministry of Health)	Facility staff
	Trainer of Trainees	Stakeholders engaged in health interventions (health actors)	CHEW
			CHW
			Community Health Committee
Who should be decision maker on when to use information	Health facility with support from sister (catholic)	Health officer (Ministry of Health)	CHEW
		household heads	Community Health Committee
Right time to use data	Immediately after collection	Immediately feedback is given	Immediately feedback is given
		At beginning of the year	
Plan to use available data	In 3 weeks	After Ministry of Health gives feedback on the data collected	According to community needs
		When Ministry of Health gives go ahead	
		When government makes announcements on major health topic to be addressed	
		According to community needs	
Changes in health due to use of data	Increased use of antiretroviral drugs for HIV infection	Decrease in infant deaths	The system is effective since the information provides evidence base for health status improvement
	Increased immunization uptake	Increased use of family planning methods	
		Improvement in environmental health	
		CHW training has been encouraged	
		Improved hygiene	
		Increased response to HIV/AIDS interventions and reduced stigma	
		Improved health practices, e.g. breast-feeding	

**Practice of health workers on information use for continuous health improvement**: There were contrasting responses among the CHWs on whether the community conducted dialogue during which data could be used to guide the dialogue process. The Community Health Committee representatives and the CHEWs indicated that dialogue was conducted while some of the CHWs contradicted this information. When dialogue was conducted, the participants included HIV-positive clients, women and youth groups, MOH representatives, nurses, CHWs, representatives of community based organizations and caregivers of orphans and vulnerable children (Table 4). The last dialogue that was held discussed support of HIV patients, health education on STIs among the youth and importance of exclusive breastfeeding.

**Table 4:** Practice on use of information for continuous improvement of health service delivery

	Chairman	Community Health Workers	Community Health Extension Workers
**Dialogue conducted in the community**	Yes	Yes	Yes
		No	
Participants in the session	Clients (HIV), youth groups, CDC water projects, MOH representatives, catholic sisters	Community Health Workers, MOH representative, youths, women groups, assistant chief, Community based organizations, APHIA Nyanza, mothers, caregivers of orphans and vulnerable children	Community members, Partners, Community Health Extension Worker, CHW, Community Health Committee
Summary of conclusion of last dialogue	Support of HIV patients	HIV & AIDS and support given	Low uptake of vitamin A, need for outreach to the community
		STIs among the youth	
		breastfeeding	
Data source	Data from household visits	Ministry of Health office, KUAP	
Lessons learnt in using data for dialogue	Data is very necessary in improvement of health status	Data brings improvement in health indicators e.g. immunization, antenatal care	It is effective, it has improved the referral system
		Improves knowledge and attitudes of mothers on health practices e.g. sanitation	
Good examples	HIV/AIDS support		
Challenges	Referrals not taken seriously by clients	Community don’t take the issues seriously, have negative attitude	Poor reporting and validity of data
		Disclosure is difficult, rejection from the community	
		Poverty that hinders implementation of practices	
		Perceptions towards Community Health Workers	
Increase of use of data (what should be done)	Trainings in data collection for the Community Health Workers.	Training Community Health Workers on data collection	Continuing training, Training the Community Health Workers on data collection, Train Community Health Committee on manual data analysis, Operations research at community level for intervention
		Involving the youth.	
		Training more people	
		Data should be organized in a language understandable to the community	
		Provide incentives for CHWs	
What is currently being done to increase the use of information?	Introduction of an identity badge for CHWs to make it easier to collect data.	Attending training which does not require payment	
		Encouraging community to attend dialogue,	
		Household visits	
How else can data be used?	Continuous training on health issues	To provide technical support or human resource	
		Ministry of Health take active role in data collection	
		Through CHWs, assistant chiefs, community support groups	

*Health System challenges in information utilization*: lack of resources and harmonized tools were mentioned as barriers to information access and utilization in the community by the CHEWs and the facility in charges, while weak linkages and coordination between the facility and community and with other partner organisations were mentioned largely by the CHWs as hindrances to information access and use. The CHWs mentioned poor coordination between them and the CHEWs, especially in referrals. The staff at the health facility were perceived to handle clients poorly, discouraging them from return visits for services: “When one is referred to the health facility, how they are handled makes them defaulters”, reported a CHW. A major system challenge was the unavailability of data tools. “Reporting tools are delivered late making the (data collection) exercise hard, responses from the facility are not up to date and are often negative to the Community Health Worker”, a view cited by a CHW and shared by the Community Health Committees. Other challenges included lack of regular attendance in dialogue sessions by community members and untimely use of collected information. The validity of the collected data collected was also not assured as perceived by the CHEWs and community health committees. These barriers were cited as making it difficult to organize or plan for dialogue and outreaches. The barriers also made it difficult to relate individuals' disease incidences recorded at the facility, with the individuals' locations in community.

**Health worker personal challenges in information utilization**: Challenges that were personal to the health workers were mentioned as barriers to information utilization. A major challenge was the lack of knowledge on data analysis and interpretation. Some community members were not sure of the accuracy and completeness of the data collected by CHWs as cited by a member of the CHC who said “refresher course(s) for CHWs on valid and reliable data (should be done), so that the data collected reflects the true situation on the ground”. The CHWs also cited lack of motivation and high workload as exemplified by the following quotes from CHWs: *“respondents from households have high expectations, saying we use their names to eat (benefit). Which creates enmity between the community and CHWs”; “there is also no motivation during data collection for CHWs yet the workload is too much (100 households for each CHW)”; “we need motivation like bags, gumboots, raincoat, and some cash monthly and pain killers to carry during household visits”*. CHWs reported challenging experiences where communities were not always receptive to health messages. Disclosure of HIV status was difficult while poverty in the community hindered implementation of health practices. Other challenges unique to the CHWs and the work they do were high expectations from the community, where households expected much from them during household visits and some had grown tired of responding to questions. There were also myths and misconceptions about health issues, for example on family planning; on use of mid-upper arm circumference tapes for nutrition assessments. Some household members kept migrating from one area to the next, making it difficult to maintain registers.

## Discussion

**Statement of principal findings**: Data appeared to play a key role in disseminating health information through community dialogue sessions and for planning and monitoring service delivery. The availability of information and awareness of persons able to conduct dialogue in the community implies that the health workers and stakeholders interviewed knew and were aware of their responsibility in data utilization. However, the lack of data use by peripheral health facilities points to the weak links in the information system and the need to enforce health information utilization between peripheral facilities and referral sites. Effective use of information is partly dependent on reliable information sources. While the major information sources emanated from the health system, the harmonization of their use was found to be lacking, resulting in non-use, leading to possible duplication or contradiction with other non-formal information sources. The lack of convergence in the dialogue topics and the content of the CBHIS may have compromised the services offered to communities. CHWs may focus on certain topics due to their perceptions on health priorities but which may not be in line with data in the CBHIS or with priority health needs of communities. The differing perceptions on who should authorise use of health information and who should engage with the information as part of dialogue processes with communities indicate inadequate information utilization. Clearly the use of information for planning and decision-making was not a culture among community level respondents. Similarly, the divergent responses on who should decide and use information for dialogue presented a disconnected scenario on the perceived and actual responsibilities of the health workers and CHWs. There appeared to be a gap between having information and its use for community dialogue and household uptake.

Furthermore, there was a lack of timely use of information which may have led to haphazard planning and health interventions that were unrelated to household health needs. The information-seeking behaviour and use of information by the respondents interviewed was poor while most of the respondents did not own the data available at the community level. Increasing the number of dialogue days from one to two days in a month was suggested by CHEWs, CHWs and CHC as a strategy for improving data use and encouraging community attendance. The frequency of dialogue and interactions between the key users of data may lead to greater use of health information among them. Frequent household visits by CHWs accompanied by correct household data collection as part of monthly reports can enhance data use at community levels and strengthen the health information linkage with health facilities. The use of role plays derived from community data to act out situations in the community is an entertaining and vibrant mode of communication and is likely to lead to health information uptake during dialogue sessions.

The health systems challenges arising from lack of coordination between the CHWs and the CHEWs may impact on the referral system which eventually takes a toll on the community and facility relationships. Poor relationships between the community and the health facility impacts on health service utilization. Timely delivery of data collection tools to the health workers can enhance planning for dialogue and improve updates of the chalk board community information. Training in data collection and manual analysis at the community level was cited as one way to increase demand and use of data and may address gaps in data use that has been identified by other researchers [5]. Training of CHEWs and CHWs in simple analytical and interpretation techniques coupled with monitoring and evaluation of the collected can lead to confidence in the data and its use for dialogue and planning.

**Strengths and weaknesses of the study**: Being a purely qualitative study was both a strength and weakness because we were able to elicit rich information on reasons why information is not being used adequately. However, the responses might not be generalizable to other settings.

**Study in relation to other studies** : Some studies, for example [14, 15], focussed mainly on utilization of the routine health information systems at the facility level. Royle majorly looked at nurses as the individuals using information. This study in contrast, examined use of information at community levels. Nzioka and Royle mainly dwelt on strategies to close the information utilization gap without addressing other areas such as sources of information and who used the information. A study in Nigeria [16] documented similar recommendations on ways to improve information utilization. Cited among the recommendations was the capacity development of volunteers to understand and appreciate the importance of reporting. Others included: 1) strong support system (community groups, schools etc.) to ease volunteers' burden; 2) supportive supervision to improve their performance; and 3) funding to motivate volunteers. Therefore issues of training, supervision and motivation cut across different studies as ways to improve information utilization. Utilization of information can be categorized in two ways. The use of commissioned research data or information for policy or practice and the use of routine data for the same. While the use of routine data has been limited to facility based data, this study expands this principle to include the use of community based data. Most utilization studies have dwelt on the use of research data or routine health facility data as opposed to community based data. Utilization of community based data comes with its own challenges because of the nature of data collectors and the linkage between the community and the health system. Data validity and reliability, which has been cited as one of the main barriers to information use by communities is not a hindrance in research or routine health facility data. While most research studies attempt to influence change at higher levels in the health systems hierarchy, the main action point for community based information is to influence change at the lowest level in the health system.

**Meaning of the study**: This study examined the perception of various stakeholders at the community level on information needs and use. By understanding the information needs of communities, the study has highlighted issues and challenges that need to be addressed by the health system. This study therefore is important to the ministry of health in its implementation of the community health strategy that aims to increase health uptake country-wide.

**Unanswered questions and future research**: One of the questions this research was not able to answer due to the scope was: what is the ideal package for motivating CHWs in order to improve information use?

## Conclusion

There is need to engage the different stakeholders at community and facility levels in order to harmonise information sources for service delivery. Dialogue processes are means for health workers to reach communities with health information, therefore dialogue topics should be based on accurate data collected from reliable sources of information. Clarify on roles of community volunteers and health facility workers on information collection will ensure timely use of data for planning and dialogue. The CHWs are the wheels that drive information use at community levels, therefore building their capacities in data collection and analysis is one way of motivating them and also improving information utilization. Improvements in linkages and coordination between the community and the health facility will lead to improved data and service utilization and eventually contribute to improved health status of the community.

### What is known about this topic

- Community health workers are able to collect valid and reliable data;
- The data available at the community level are reported onwards but underutilised in the community.

### What this study adds

- Challenges leading to underutilization of data at community level;
- Insight into the activities the health workers that may lead to the utilization of data at the lower levels of the health system in Kenya.

## Competing interests

The authors declare no competing interests.

## References

[1] Lærum Hallvard, Karlsenand Tom H, Faxvaag Arild (2004). Use of and attitudes to a hospital information system by medical secretaries, nurses and physicians deprived of the paper-based medical record: a case report. BMC Medical Informatics and Decision Making..

[2] World Health Organization (1998). World health report 1998: life in the 21st century. A Vision For All.

[3] World Health Organization (2003). The Challenge of Implementation: District Health Systems for PHC..

[4] Ball MJ, Lillies JC (2000). Health Information Systems: challenges for the 21st Century. ACN Clinical issues..

[5] Braa Jørn, Heywood Arthur, Sahay Sundeep (2012). Improving quality and use of data through data-use workshops: Zanzibar, United Republic of Tanzania. Bulletin of the World Health Organization..

[6] World Health Organization (2004). Developing health management information systems. A practical guide for developing countries..

[7] de Savigny Don, Kasale Harun, Mbuya Conrad, Reid Graham (2008). In focus: fixing health systems.

[8] DFID health resource center (2011). Health Management Information Systems.

[9] Stansfield Sally K, Walsh Julia, Prata Ndola, Evans Timothy (2006). Information to improve decision making for health, disease control priorities in developing countries.

[10] Gething Peter W, Noor Abdisalan M, Goodman Catherine A, Gikandi Priscilla W, Hay Simon I, Sharif Shahnaaz K, Atkinson Peter M, Snow Robert W (2007). Information for decision making from imperfect national data: tracking major changes in health care use in Kenya using geostatistics. BMC Medicine..

[11] Government of Kenya (2002). National Development Plan 2002-2008..

[12] Eakin Elizabeth Gand, Strycker Lisa A (2001). Awareness and barriers to use of cancer support and information resources by HMO patients with breast, prostate or colon cancer: patient and provider perspectives. Psycho-Oncology..

[13] Weber Robert Philip (1990). Basic content analysis: quantitative applications in the social sciences. Sage University Papers..

[14] Solomon Nzioka M (2005). Health information generation and utilization for informed decision-making in equitable health service management: the case of Kenya partnership for health program. International Journal for Equity in Health..

[15] Royle Joan, Blythe Jennifer (1998). Promoting research utilisation in nursing: the role of the individual, organisation and environment. Evid Based Nurs..

[16] Routine Health Information Network (2003). Second International RHINO Workshop: Enhancing the Quality and Use of Routine Health Information at District Level..

